# Standardization of Lung CT Number Using COPD Gene2 Phantom Under Various Scanning Protocols

**DOI:** 10.3390/s25092906

**Published:** 2025-05-04

**Authors:** Hoondong Song, Hanjoo Jang, Jongduk Baek

**Affiliations:** 1 School of Integrated Technology, Yonsei University, Incheon 21983, Republic of Korea; hoondongstar@yonsei.ac.kr; 2 Department of Artificial Intelligence, College of Computing, Yonsei University, Seoul 03722, Republic of Korea; imhanjoo@naver.com; 3 Bareunex Imaging Inc., Incheon 21998, Republic of Korea

**Keywords:** computed tomography (CT), CT number standardization, chronic obstructive pulmonary disease (COPD), COPD gene2 phantom

## Abstract

Lung computed tomography (CT) images are widely used to diagnose chronic obstructive pulmonary disease (COPD) by evaluating signs of lung tissue destruction. Accurate diagnosis requires standardizing the CT numbers in lung CT images to distinguish between normal and damaged tissue. The CT number standardization method proposed by Chen-Mayer et al., which uses the linearity of Martinez’s formula, showed promising results in phantom studies. However, our findings reveal that the CT number of water varies significantly, depending on scanning conditions and the characteristics of its container, making it an unreliable reference for lung CT number standardization. To enhance the standardization method, we modified the approach to exclude water and used only solid foams from the COPD gene2 phantom as references. To evaluate the proposed method, we collected 234 CT images of the COPD gene2 phantom from 8 different CT scanners and assessed performance by analyzing CT number standard deviations and variations. The modification resulted in improved reliability and consistency in CT number standardization. Additionally, for a detailed analysis, we segmented the dataset based on CT dose index (CTDI), X-ray tube potential, and reconstruction algorithms to examine the impact of different scanning protocols on standardization performance.

## 1. Introduction

Chronic obstructive pulmonary disease (COPD) is a complex lung disease that is characterized by the destruction of lung tissue and persistent airflow obstruction [1]. COPD causes breathing difficulties resulting in pulmonary emphysema, which can contribute to the development of additional respiratory complications [2]. These findings highlight the critical importance of early diagnosis for COPD. Lung computed tomography (CT) analysis is a key tool for assessing lung conditions and diagnosing COPD. This diagnostic approach leverages the contrast in CT numbers between air and lung tissues, distinguishing air spaces from damaged lung tissue [3,4,5]. By calculating the ratio of functional lung tissue to air spaces, lung CT image analysis helps diagnose COPD and evaluate its severity. The Lung Density Committee of the Quantitative Imaging Biomarkers Alliance (QIBA) has established official CT number thresholds to differentiate lung tissue from air spaces for diagnosing COPD [3]. However, the technique has limitations that restrict its ability to accurately demonstrate air-tissue contrast and produce reliable diagnostic results. A primary challenge is the inconsistency of lung density measurements, which are significantly influenced by the CT scanner manufacturer and the scanning protocols employed. In lung CT image analysis studies, images are often acquired using varying dose levels and reconstruction kernels, depending on the patient’s condition and the available scanners [4]. These variations affect the stability of lung tissue visibility and CT number measurements, thereby reducing the diagnostic accuracy of lung CT images.

**Figure 1:**
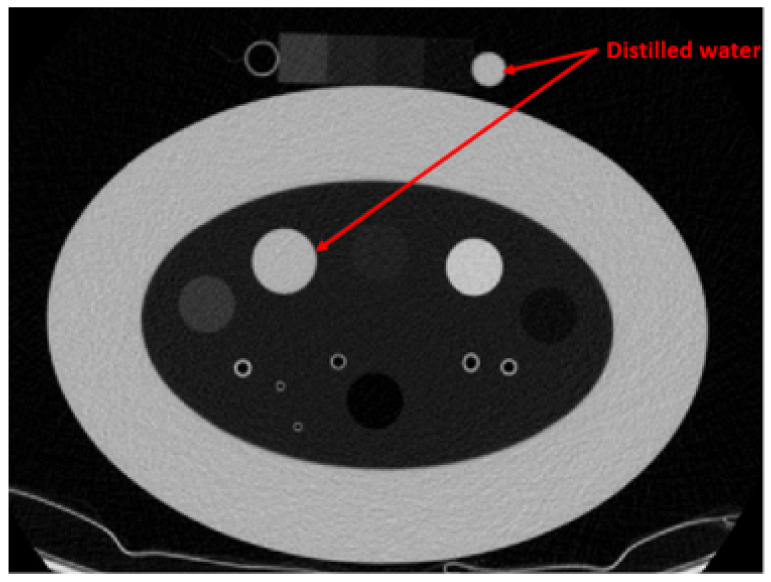
CT image of the COPD gene2 phantom with an additionally attached phantom scanned using Revolution 64 with 1.5 mGy dose. The window level is set to [0-1024] HU.

In recent years, several approaches have been proposed to address the limitation of lung CT analysis, aiming to mitigate the effects of lung density variations caused by different acquisition protocols and increase CT number consistencies. Several studies have employed the ComBat CT number standardization algorithm, which evaluates X-ray dose and acquisition protocols to scale the CT numbers based on various statistical models [6,7]. However, the ComBat technique requires parameter analysis prior to the CT number standardization, and the Lung Density Committee provides the scaling information for limited CT scanners and scanning protocols. Chen-Mayer et al. introduced lung CT density standardization method based on the Martinez’s CT number-electron density relation [8]. The method employs the COPD gene2 phantom, which contains various foams within the lung density range, as references (3 foams, air, and water) for CT number standardization. The study demonstrated the effectiveness of the method using 22 CT images acquired with different scanning protocols across four CT scanners. However, using water as a reference decreases the reliability of the standardization process. Water’s density and corresponding CT number can fluctuate due to the external influences, including bubble formation induced by CT scanner vibrations, precipitate accumulation from corrosion within the containing cylinder, and other environmental factors such as pressure and temperature. As illustrated in Figure 1 and Table 1, the average CT number of water, which is expected to be 0 Hounsfield Unit (HU) shows inconsistency even when the same scanner and scanning protocol are used.

In this study, we alternatively used a solid material instead of the water as references to improve the performance and reliability of the CT number standardization method. To assess the performance of the proposed method and applicability across various CT scanners and scanning protocols, we compared the standardization performance of the proposed method with the original approach. To explore the standardization performance for broader applicability, we used 234 CT images of the COPD gene2 phantom acquired from various CT scanners and scanning protocols, and analyzed the impact of the scanning parameters, such as dose level, tube potential and reconstruction algorithm.

## 2. Materials and Methods

### 2.1. COPD Gene2 Phantom

**Figure 2:**
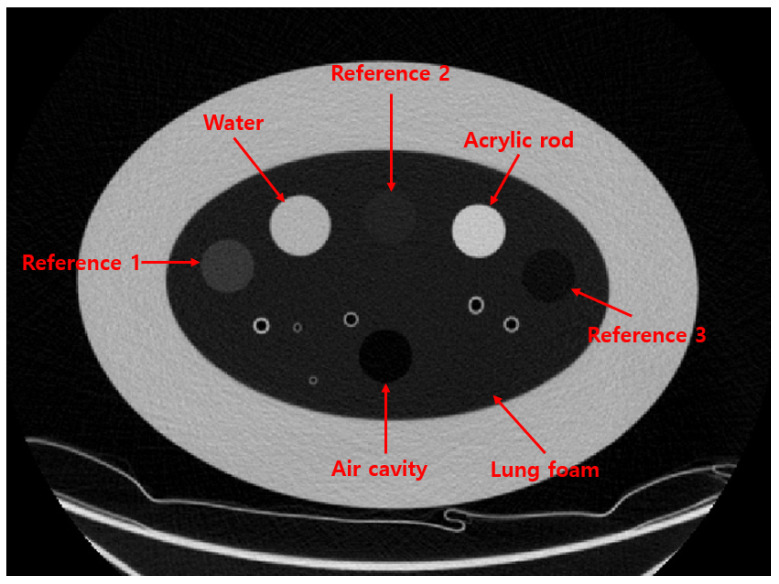
CT image of COPD gene2 phantom.

To evaluate the performance of the proposed method, we used the COPD gene2 phantom (CTP698; The Phantom Laboratory, Schenectady, NY, USA), as shown in Figure 2. The phantom comprises air and water sealed in silicone cylinders, an acrylic rod, and three reference foams with different densities. The nominal densities of the reference foams are 64.2 kg/m^3^, 192.6 kg/m^3^, and 321.0 kg/m^3^, respectively [9,10]. These reference foams are surrounded by an oval-shaped lung foam with a measured density of 160.2 kg/m^3^, corresponding to normal pulmonary parenchyma [10].

All materials in the COPD gene2 phantom are registered with the National Institute of Standards and Technology (NIST) Standard Reference Material (SRM) foam suite [11,12]. Based on the SRM foam suite, the CT numbers of the reference foams are expected to be −700, −820, and −935 HU, respectively, while the lung foam has a CT number of −856 HU. A detailed list of the materials and their corresponding densities and CT numbers in the COPD gene2 phantom is provided in Table 2.

### 2.2. CT Number Standardization

The CT number standardization method consists of two primary steps: internal calibration and monochromatic energy mapping. During the internal calibration step, the CT numbers of the reference foams and the lung foam are calibrated using the linear relationship between their measured CT numbers and nominal densities. The internal calibration step serves to reduce the initial intra-variability among the reference foams, thereby improving the accuracy of the standardization process. While Chen-Mayer’s study used air (−1000 HU) and water (0 HU) as calibration references, we excluded water and instead used reference foam 1 (−700 HU) as an alternative calibration basis. The offsets for the remaining reference foams, $\Delta_{r}$, in the COPD gene2 phantom are calculated as follows:

(1) $$\Delta_{r}=\left(\delta_{r1}-\delta_{a}\right)\frac{\rho_{f}}{\rho_{r1}}+\delta_{a}$$

where $\delta_{r1}$ and $\delta_{a}$ represent the offsets for reference foam 1 and air, respectively, calculated by subtracting their measured CT numbers from −700 and 0 HU. $\rho_{r1}$ and $\rho_{f}$ are the electron densities of reference foam 1 and the other reference foams. The electron densities of each foam are calculated using the NIST SRM foam suite [13,14]. To calculate the true CT number of the foam 1, we used prompt gamma neutron activation analysis (PGAA) of the foam composition, which is provided by the Neutron Research Center of NIST [12]. The measured CT numbers of the other foams were then calibrated by subtracting the calculated CT number offsets as follows:

(2) $$H{\left(r\right)}_{cal}=H{\left(r\right)}_{raw}-\Delta_{r}/1000$$

where $H{\left(r\right)}_{cal}$ and $H{\left(r\right)}_{raw}$ represent the rescaled and shifted CT numbers corresponding to the calibrated and measured values for each foam, respectively. Specifically, the rescaled and shifted CT number is defined as CTnumber/1000+1. The internal calibration step ensures that $H{\left(r\right)}_{cal}$ falls within the range of 0 to 1, facilitating the application of Martinez’s formula and reducing numerical errors in the estimation of standardized CT numbers.

In the second step, the energy mapping process utilizes Martinez’s single scanner-dependent parameter formula, which is a simplified version of Schneider’s multi-parameter formula [15]. Schneider’s formula separately accounts for the coherent, incoherent, and photoelectric components of X-ray attenuation. Assuming a linear relationship between the electron density and the CT number of scanned materials, Martinez’s formula combines these attenuation components into a single parameter, α(r), specific to each scanner and scanning protocol, as follows:

(3) $$H{\left(r\right)}_{cal}/\rho_{e}*=\alpha \left(r\right)\left(1-Z_{eff}{*}^{n}\right)+Z_{eff}{*}^{n}$$

where $H{\left(r\right)}_{cal}$, $\rho_{e}*$, and $Z_{eff}*$ represent the rescaled CT number, the ratio of electron density to that of water, and the effective atomic number, respectively. According to the SRM suite of the COPD gene2 phantom, $\rho_{e}*$ and $Z_{eff}*$ are defined as 0.956 and 0.871, respectively, for thoracic soft tissues [13]. The parameter *n*, an atomic constant associated with low-density materials, is estimated to be 3.21 for materials with densities below 1000 kg/m^3^ [14]. Using Equation (3), α(r) for each reference foam and air can be calculated. Based on the assumption of Martinez’s formula, the scanner-dependent parameter $\bar{\alpha }$, which is uniquely determined for each scanner and acquisition protocol, is estimated using linear regression of α(r) calculated from the three reference foams and air. Using the estimated $\bar{\alpha }$, the scanner-independent electron densities of the calibration target, $\rho_{e,t}^{*}$, for the target foam are calculated as follows:

(4) $$\rho_{e,t}*=\frac{H(t)}{\bar{\alpha }\left(1-Z_{eff}{*}^{n}\right)+Z_{eff}{*}^{n}}$$

where H(t) is the measured CT number of the standardization target (lung foam). Using the estimated electron density, the CT number of target is calibrated as follows:

(5) $$H_{80}\left(t\right)=\rho_{e,t}*\left[\alpha_{80}\left(1-Z_{eff}{*}^{n}\right)+Z_{eff}{*}^{n}\right]$$

where $H_{80}\left(t\right)$ represents the rescaled CT number of the target calibrated to a monochromatic energy level of 80 keV, and $\alpha_{80}$ is the scanner-dependent parameter at 80 keV, pre-determined to be 0.946. In this study, we chose 80 keV as the standardization energy level because the CT numbers measured at this energy level roughly corresponds to the range of CT numbers observed at 120 kVp CT spectrum. The final standardized CT number, $CTnumber_{80}\left(t\right)$, is calculated by inversely scaling and shifting the $H_{80}\left(t\right)$ value with $(H_{80}\left(t\right)-1)1000$.

### 2.3. Data Acquisition

To evaluate the effectiveness of the proposed method, the COPD gene2 phantom was scanned using 4 Siemens (Siemens Healthineers, Erlangen, Germany), 3 GE (GE healthcare, Chicago, IL, USA), and 1 Philips (Philips Healthcare, Andover, MA, USA) scanners. Table 3 provides a summary of the CT scanners and corresponding scanning parameters. For each scanner, different scanning protocols, the CT dose index (CTDI), tube potential, and reconstruction algorithm, are used to create a dataset. The details of each protocol are as follows:

(1) CT Dose Index (CTDI) Setting: Low-dose CT scans are often performed to minimize radiation exposure to patients. However, reducing the dose increases image noise, which in turn amplifies CT number variability [16,17]. To evaluate the impact of dose levels on standardization performance, we scanned the COPD gene2 phantom using 4 different CTDI settings: 1.0, 1.5, 3.0, and 6.0 mGy.

(2) Tube potential setting: At a fixed dose level, CT numbers can vary, depending on the tube potential, as it determines the energy level of photons emitted by the X-ray source [17]. To investigate this effect, we analyzed CT number variation and its impact on standardization performance across different tube potentials: 80, 100, and 120 kVp. Note that protocols with 1.0 mGy CTDI are conducted with 80 and 100 kVp, while protocols with 6.0 mGy are conducted with 120 kVp only.

(3) Reconstruction algorithm setting: CT image reconstruction algorithms and utilized kernels affect the image sharpness and noise characteristics [18]. For this study, we reconstructed the CT images with 4 different reconstruction algorithms; filtered back-projection (FBP) with standard and sharp kernels and iterative reconstruction (IR) with 100% and 40% regularization strengths. As demonstrated in Table 3, we classified the kernels that do not impact the CT number contrast as “standard kernel” and those that enhance edge contrast as “sharp kernel”.

For each reconstruction algorithm, 9 combinations of CTDI levels and tube potentials were used and total 234 CT images of COPD gene2 phantom are collected. For certain CT vendors, the use of a CT number truncation filter, designed for image capacity compression, can artificially elevate the CT number of air. To prevent errors caused by this truncation, we applied a truncation correction step prior to performing CT number standardization [19].

To obtain the CT numbers of the reference foams and the lung foam in the CT images of the COPD gene2 phantom, we averaged the values from a 5×5×5 voxel region at the center of each foam in the phantom. Using these extracted CT numbers, the CT numbers of the lung foam in each CT image of the COPD gene2 phantom were standardized across different scanners and scanning protocols.

## 3. Results

To assess the performance of the proposed standardization method and investigate the effect of each protocol on the standardization performance, we calculated the mean and standard deviation of the CT numbers across various scanners and protocols, both before and after standardization. We labeled the stages as “Raw” (before standardization), “Internal Cal.” (after internal calibration), and “Standardization” (after monochromatic energy mapping) in this section. A reduction in the standard deviation of the CT numbers reflects a reduction in CT number variation and the effectiveness of the standardization process. To visualize CT-number distributions and compare variations at each step of standardization, box plots of CT numbers are provided using the reference foams and the lung foam.

To evaluate the overall effectiveness of the proposed standardization method and the impact of using water as a reference, we statistically analyzed the CT numbers of reference foams 2 and 3, as well as the lung foam, across all 234 datasets.

**Figure 3:**
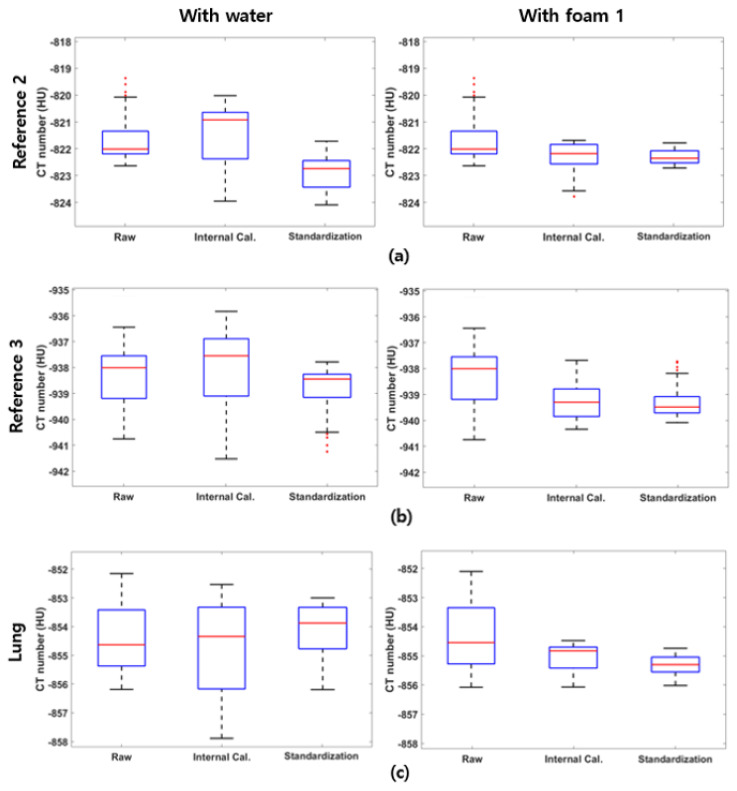
Box plots of the standardization steps for (**a**) reference foam 2, (**b**) reference foam 3, and (**c**) lung foams. The left column displays the standardization results using water as a reference, while the right column shows the results using foam 1 as a reference.

As shown in Table 4, using water as one of the standardization references increases the standard deviation of the reference foams and the lung foam during the internal calibration step. This might be caused due to the CT number uncertainty of the water as a standardization reference. Since water serves as the calibration basis during the internal calibration, the CT number fluctuation of water propagates to other reference foams and the lung foam during the calibration. On the other hand, alternating the reference foam 1 as a basis prevents the error during the internal calibration for all foams, as demonstrated in Table 5. The standard deviation of CT numbers for each foam decreases at each step, demonstrating the effectiveness of the proposed method. Additionally, narrowing the standardization density range from [0, 1000] kg/m^3^ to [0, 321.0] kg/m^3^ improves the validity of the linearity assumption between electron density and CT number, resulting in enhanced performance, as illustrated in Figure 3.

Although the overall standardization analysis confirmed the effectiveness of the proposed method, the dose level during the CT scan has a significant impact on its performance. As shown in Table 6, the standardization results for each dose level indicate that performance varies with the noise level in the CT images. While the mean CT number at each dose level remains unaffected, the standard deviation tends to increase under low-dose conditions. At 1.0 mGy, the elevated noise level amplifies CT number variations for the foams in the COPD gene2 phantom, diminishing their reliability as reference materials.

Table 7 provides an analysis of standardization performance across different reconstruction algorithms and kernels. Sharp kernels in FBP enhance tissue contrast but also increase noise levels, leading to amplified CT number variation for the references and reduced standardization performance. As shown in Table 7, FBP with sharp kernels exhibited the highest standard deviation and the lowest reduction rate after standardization due to the noise enhancement. In contrast, IR demonstrated superior performance in CT number standardization. By reducing image noise during reconstruction, IR achieves a similar effect to increased scanning doses, minimizing image noise and CT number variation in the reference foams. Notably, at lower dose levels, CT images reconstructed with IR showed better standardization performance than those reconstructed with FBP, largely due to IR’s effective noise reduction capabilities. However, IR compromises image visibility by excessively smoothing low-level features and reducing the standardization performance. Demonstrated in Table 7, IR with 40% regularization strength reduced the CT number standard deviation from 1.56 to 0.31, whereas 100% regularization achieved a more modest reduction from 1.47 to 0.46.

To further investigate the impact of tube potential on CT number standardization performance, we categorized the data by tube potential and standardized the CT numbers of the lung foam. As shown in Table 8, higher tube potentials resulted in lower standard deviations of the CT numbers for the lung foam, indicating improved standardization performance. Increasing the tube potential reduces CT number variation by enhancing the signal-to-noise ratio in CT images and minimizing photon starvation as the X-rays pass through the scanned object.

However, increasing the tube potential does not always lead to better standardization performance. As shown in Table 9, at a dose level of 1.0 mGy, the reduction in CT number standard deviation is greater at 80 kVp compared to 100 kVp. Similarly, at 1.5 mGy, 100 kVp demonstrated the best performance for CT number standardization, reducing the standard deviation to 0.74 from 2.22. This variation occurs because tube potential and tube current are inversely proportional at a fixed dose level. Increasing the tube potential decreases the X-ray flux from the source, while low tube potential can result in radiation saturation due to insufficient photon energy. On the other hand, low tube current increases noise levels due to a reduced number of photons.

To optimize CT number standardization performance, maintaining a balance between tube potential and current is critical. This balance will vary, depending on the total CTDI used for scanning and patient-specific characteristics, such as size, density, and tissue composition.

## 4. Discussion

CT numbers can vary, depending on the scanner and scanning protocol, making it essential to follow a specific protocol to obtain reliable CT images for COPD diagnosis [20,21,22]. However, this requirement limits the ability to establish universally accepted CT number thresholds for COPD. To overcome this challenge, Chen-Mayer introduced a standardization method based on scanner-dependent parameters to reduce variations in CT lung density measurements. Unlike traditional diagnostic approaches, this method allows for the establishment of protocol-independent thresholds for COPD diagnosis.

In this study, we expanded the analysis to include a wider range of scanners and protocols by examining 234 CT images of the COPD gene2 phantom. This broader evaluation enabled us to assess the effectiveness of the standardization method across diverse scanners and scanning protocols, including variations in CTDI, tube potential, and reconstruction algorithm settings. Building on Chen-Mayer’s CT number standardization method, we excluded water from the reference list to narrow the effective density range and eliminate the variability associated with liquid materials. This modification improved the reliability of the reference materials and reduced CT number variation in the images, leading to enhanced standardization performance. As a result, the standard deviation of CT numbers for the lung foam decreased to 0.97 from 2.12, demonstrating consistent performance across a broad range of scanning protocols.

In conventional studies and clinical workflows, water is frequently used as a reference material for imaging calibration and CT number standardization. However, our findings indicate that the CT number of water is prone to variability due to several factors, including bubble formation induced by CT scanner vibrations, precipitate formation from corrosion within the water-containing cylinder, and other environmental effects. To improve the robustness of the standardization process, we substituted water with foam 1, a solid reference material included in the COPD gene2 phantom.

The use of foam 1 not only mitigated CT number fluctuations but also enhanced the consistency and accuracy of the standardization by constraining the calibration density range to [321.0 1000] kg/m^3^. This narrower range helps minimize approximation errors associated with the parameter simplification in the Martinez formula, which models the relationship between CT number and electron density through a single attenuation parameter, $\bar{\alpha }$. While reducing the density range improves estimation precision, the performance gain eventually saturates, and the calibration range must still encompass the density of the target material. Therefore, foam 1 was chosen as a practical and effective reference material for this study.

Additionally, the effects of the dose level, tube potential and reconstruction kernel on the performance of the standardization procedure are analyzed as follows:

(1) CT number variation increases at low dose levels due to elevated image noise, which negatively impacts the performance of the standardization process under such conditions. However, applying noise reduction techniques can mitigate this performance degradation. For instance, the noise-suppressing capabilities of IR improved standardization performance, even at low dose levels.

(2) In general, increasing the tube potential reduces CT number variation by enhancing the signal-to-noise ratio in CT images and minimizing photon starvation as X-rays pass through the patient. However, at a fixed dose level, higher tube potential decreases the X-ray flux, which can impact image contrast and noise levels. CT numbers also vary, depending on the tube potential of the X-ray source. In the diagnosis of COPD using CT lung density measurements, the choice of tube potential varies based on dose level and patient-specific thoracic characteristics, such as size, density, and tissue composition. Based on the results, it is crucial to strike a balance between tube potential and X-ray current to optimize the performance of CT number standardization.

(3) CT image sharpness and noise levels vary, depending on the reconstruction techniques used. FBP with a sharp kernel exhibited notable degradation in standardization performance due to increased image noise accompanying contrast enhancement. At low dose levels, CT images reconstructed with IR demonstrated better standardization performance than those reconstructed with FBP, primarily because of IR’s effective noise reduction capabilities. However, excessive regularization strength in IR can compromise image visibility in clinical CT images, potentially reducing diagnostic accuracy.

To implement the proposed method to clinical CT images, several validations must be considered. We found the strong dependency of the standardization performance on CT noise levels, and the noise-reducing characteristics of IR increase the performance of the proposed method. Given the higher image noise from low-dose scanning in clinical environments, noise reduction techniques are expected to have a more pronounced effect on improving standardization performance. However, to ensure the robustness of the CT number standardization and corresponding diagnostic performance, it is important to optimize the denoising strength in order to prevent the reduction in CT number contrast, considering the complex anatomical tissue structure. In future work, we plan to investigate the impact of advanced noise reduction techniques, such as Block-Matching 3D filtering (BM3D) and machine learning-based denoisers, on CT number standardization.

The primary objective of the proposed method is to standardize CT numbers of lung tissues to improve the diagnostic accuracy of COPD using lung CT images. As the method only requires reference materials scanned under the same imaging protocols, it can be seamlessly integrated into existing clinical CT imaging and diagnostic workflows without altering current acquisition procedures. The reference materials may be miniaturized and integrated onto the patient table, thereby facilitating the application of the proposed method directly to patient CT images without necessitating an additional reference phantom scan. With further clinical validation using patient CT data, the method holds promise for incorporation into CT image reconstruction systems and diagnostic software for COPD assessment.

In conclusion, we proposed and evaluated a novel method for standardizing the CT number of lung foam in the COPD gene2 phantom. The proposed method reduces diagnostic dependency on CT scanners and scanning protocols, enhancing reliability in distinguishing lung tissue from air pockets.

## Figures and Tables

**Table 1:** Average CT numbers of water in COPD gene2 phantom and attached phantom measured from five repeated scans.

Water (HU)	
COPD gene2 Phantom	Attached Phantom
1.8	−2.7
19.5	1.7
5.0	−7.8
8.9	2.6
14.7	−3.1

**Table 2:** Reference foams of COPD gene2 phantom.

Foam Number	Foam	Density (kg/m^3^)	CT Number (HU)
1	Reference 1	321.0	−700
2	Water	1000.0	0
3	Reference 2	192.6	−820
4	Acrylic rod	1180.0	130
5	Reference 3	64.2	−935
6	Air	0	−1000
7	Lung	160.2	−835

**Table 3:** Summary of scanner and scanning protocols.

Vendor	Scanner	Detector	Slice Thickness	Reconstruction Kernel
		Collimation	/Interval (mm)	
Siemens	Force	64 × 0.6	0.75/0.5	Bf40d (Standard Kernel)
				Qr44d (Sharp kernel)
				Bf40d ADMIR 5 (IR 100%)
				Bf40d ADMIRE 2 (IR 40%)
	Definition Flash	64 × 0.6	0.75/0.5	B31f (Standard Kernel)
				B45f (Sharp Kernel)
				L31f SAFIRE 5 (IR 100%)
				L31f SAFIRE 2 (IR 40%)
	Definition AS+	64 × 0.6	0.75/0.5	B31f (Standard Kernel)
				B46f (Sharp Kernel)
				L31f SAFIRE 5 (IR 100%)
				L31f SAFIRE 2 (IR 40%)
	Sensation 64	32 × 0.6	0.75/0.5	B31f (Standard Kernel)
				B45f (Sharp kernel)
GE	750HD	64 × 0.625	0.625/0.5	Standard (Standard Kernel)
				Bone (Sharp Kernel)
				Std ASIR 100% (IR 100%)
				Std ASIR 40% (IR 40%)
	Revolution	32 × 0.6	0.75/0.5	Standard (Standard Kernel)
				Bone (Sharp Kernel)
				Std ASIR 100% (IR 100%)
				Std ASIR 40% (IR 40%)
	VCT	32 × 0.6	0.75/0.5	Standard (Standard Kernel)
				Bone (Sharp Kernel)
Philips	Brilliance iCT	64 × 0.625	0.67/0.5	YB (Standard Kernel)
				B (Sharp Kernel)
				I3, iDose (5) (IR 100%)
				I3, iDose (3) (IR 40%)

**Table 4:** Statistical results for CT number standardization with water as a reference.

	Mean ± Standard Deviation (HU)		
Foam	Raw	Internal Cal.	Standardization
reference foam 2	−822.25 ± 2.24	−821.78 ± 4.48	−822.92 ± 1.71
reference foam 3	−938.86 ± 2.51	−938.30 ± 4.14	−939.72 ± 1.87
Lung Foam	−854.29 ± 2.12	−855.14 ± 4.49	−854.66 ± 2.21

**Table 5:** Statistical results for CT number standardization with foam 1 as a reference.

	Mean ± Standard Deviation (HU)		
Foam	Raw	Internal Cal.	Standardization
reference foam 2	−822.25 ± 2.24	−822.46 ± 1.31	−822.60 ± 0.85
reference foam 3	−938.86 ± 2.51	−939.75 ± 1.03	−939.91 ± 0.81
Lung Foam	−854.29 ± 2.12	−855.28 ± 1.63	−855.25 ± 0.97

**Table 6:** Statistical results based on dose level for the lung foam in COPD gene2 phantom images.

	Mean ± Standard Deviation (HU)		
CTDI (mGy)	Raw	Internal Cal.	Standardization
1.0	−853.25 ± 2.41	−855.42 ± 1.93	−855.51 ± 1.21
1.5	−854.16 ± 2.22	−855.36 ± 1.26	−855.82 ± 0.93
3.0	−854.63 ± 1.40	−854.18 ± 1.18	−854.52 ± 0.65
6.0	−855.74 ± 1.01	−855.06 ± 0.86	−855.21 ± 0.44

**Table 7:** Statistical results based on reconstruction algorithm for the lung foam in COPD gene2 phantom images.

	Mean ± Standard Deviation (HU)		
Reconstruction	Raw	Internal Cal.	Standardization
FBP (Standard)	−854.43 ± 2.58	−855.44 ± 1.51	−855.94 ± 0.93
FBP (Sharp)	−854.18 ± 2.64	−855.85 ± 1.72	−855.52 ± 1.28
IR (40%)	−854.16 ± 1.56	−855.20 ± 0.51	−855.04 ± 0.31
IR (100%)	−854.36 ± 1.47	−854.48 ± 0.67	−854.23 ± 0.46

**Table 8:** Statistical results based on tube potential for the lung foam in COPD gene2 phantom images.

	Mean ± Standard Deviation (HU)		
Tube Potential (kVp)	Raw	Internal Cal.	Standardization
80	−853.64 ± 2.77	−855.04 ± 1.64	−855.19 ± 1.32
100	−853.91 ± 2.82	−854.95 ± 1.39	−855.45 ± 0.96
120	−855.32 ± 1.53	−854.84 ± 0.76	−855.36 ± 0.52

**Table 9:** Statistical results based on tube potential at each dose level for the lung foam in COPD gene2 phantom images.

CTDI	Tube Potential	Mean ± Standard Deviation (HU)		
(mGy)	(kVp)	Raw	Internal Cal.	Standardization
1.0	80	−853.42 ± 2.21	−855.97 ± 1.49	−855.73 ± 0.84
	100	−853.08 ± 2.87	−854.87 ± 2.17	−855.29 ± 1.46
1.5	80	−853.68 ± 2.71	−855.22 ± 1.64	−855.71 ± 1.08
	100	−854.21 ± 2.22	−855.61 ± 1.22	−855.85 ± 0.74
	120	−854.59 ± 1.88	−855.25 ± 1.01	−855.90 ± 0.77
3.0	80	−853.81 ± 2.08	−853.95 ± 1.29	−854.13 ± 0.93
	100	−854.44 ± 1.57	−854.38 ± 1.14	−854.46 ± 0.75
	120	−855.64 ± 1.22	−854.21 ± 0.98	−854.97 ± 0.62
6.0	120	−855.74 ± 0.98	−855.06 ± 0.70	−855.21 ± 0.44

## Data Availability

The data that support the findings of this study are available upon reasonable request to the corresponding author due to privacy.
